# Abnormal vascular thickness and stiffness in young adults with type 1 diabetes: new insights from cutting-edge ultrasound modalities

**DOI:** 10.1186/s12933-024-02280-5

**Published:** 2024-05-24

**Authors:** Michael J. De Biasio, Michelle Furman, Antoine Clarke, Wei Hui, Yesmino Elia, Jerome Baranger, Olivier Villemain, Luc Mertens, Farid H. Mahmud

**Affiliations:** 1 Division of Endocrinology, Department of Pediatrics, The Hospital for Sick Children, Temerty Faculty of Medicine, University of Toronto, Toronto, ON Canada; 2 Department of Pediatrics, Division of Endocrinology and Metabolism, The Hospital for Sick Children, University of Toronto, Toronto, ON Canada; 3 Division of Cardiology, Department of Pediatrics, The Hospital for Sick Children, University of Toronto, Toronto, ON Canada

**Keywords:** Type 1 diabetes, Carotid intima-media thickness, Pulse wave velocity, High frequency ultrasound, Ultrafast ultrasound imaging, Cardiovascular disease

## Abstract

**Background:**

Cardiovascular disease (CVD) remains the leading cause of morbidity and mortality in patients with Type 1 Diabetes (T1D). Early markers of CVD include increased carotid intima-media thickness (CIMT) and pulse wave velocity (PWV), but these existing ultrasound technologies show limited spatial and temporal resolution in young adults. The purpose of this study is to evaluate the utility of high-resolution ultrasound modalities, including high frequency ultrasound CIMT (hfCIMT) and ultrafast ultrasound PWV (ufPWV), in young adults with Type 1 Diabetes.

**Methods:**

This is a prospective single-center observational cohort study including 39 participants with T1D and 25 age and sex matched controls. All participants underwent hfCIMT and ufPWV measurements. hfCIMT and ufPWV measures of T1D were compared with controls and associations with age, sex, BMI, A1c, blood pressure, and lipids were studied.

**Results:**

Mean age was 24.1 years old in both groups. T1D had a greater body mass index (27.7 [5.7] vs 23.1 [3.2] kg/m^2^), LDL Cholesterol, and estimated GFR, and had a mean A1c of 7.4 [1.0] % (57 mmol/mol) and diabetes duration of 16.1 [3.7] years with 56% using insulin pumps. In T1D, hfCIMT was significantly increased as compared to controls (0.435 ± 0.06 mm vs 0.379 ± 0.06 mm respectively, p < 0.01). ufPWV measures were significantly increased in T1D (systolic foot PWV: 5.29 ± 0.23 m/s vs 5.50 ± 0.37 m/s, p < 0.01; dicrotic notch PWV = 7.54 ± 0.46 m/s vs 7.92 ± 0.41 m/s, p < 0.01). Further, there was an impact of A1c-measured glycemia on hfCIMT, but this relationship was not seen with ufPWV. No significant statistical correlations between hfCIMT and ufPWV measures in either T1D or healthy controls were observed.

**Conclusion:**

Young adults with T1D present with differences in arterial thickness and stiffness when compared with controls. Use of novel high-resolution ultrasound measures describe important relationships between early structural and vascular pathophysiologic changes and are promising tools to evaluate pre-clinical CVD risk in youth with T1D.

Trial Registration: ISRCTN91419926.

**Supplementary Information:**

The online version contains supplementary material available at 10.1186/s12933-024-02280-5.

## Background

Type 1 Diabetes is a significant risk factor for cardiovascular disease (CVD) [1]. People with type 1 diabetes (T1D) have a 2–3 times increased risk of CVD and corresponding mortality [2, 3], with an 8–10 times greater risk in cases with impaired glycemic management [4]. T1D is predominantly diagnosed at a younger age, and clinical manifestations of CVD develop insidiously and appear late in the pathogenesis of CVD [5]. The evaluation of subclinical CVD includes carotid intima-media thickness (CIMT) and arterial stiffness using pulse wave velocity (PWV), both of which have been associated with an increase in major adverse cardiac events later in adulthood [6–8]. CIMT describes the thickness of the media layer of the carotid artery, measured via 2-dimensional B-mode ultrasound along a longitudinal section of the common carotid artery [9]. However, its limited resolution has led to failures in measuring cardiovascular effects in some instances, leading to considerations for improved spatial resolution and incorporation of other markers of cardiovascular health [10]. Pulse wave velocity (PWV) is a well-studied technique evaluating arterial stiffness, a marker of vascular health and CVD risk [11]. While there are a variety of methods available to determine arterial stiffness, it is traditionally measured by determining the speed of pulse propagation between carotid and femoral vessels, termed carotid femoral PWV [11]. This global assessment of PWV has notable limitations, relying on imprecise measurements that are impacted by non-uniform vessel behaviour and wave reflections [11].

Given these limitations, two recent novel imaging modalities, high-frequency ultrasound and ultrafast ultrasound imaging, offer improved techniques to evaluate CIMT and PWV that may help identify earlier signs of pathologic changes in the vasculature of young adults with T1D [12, 13]. CIMT measured by high frequency ultrasound (hfCIMT) allows for non-invasive, high spatial resolution imaging of blood vessel structure that improves measurement and reliability of CIMT [12]. Ultrafast ultrasound utilizes high temporal-resolution acquisition to allow for local arterial PWV assessment with high accuracy, via the direct visualization of the pressure wave travelling along the arterial wall [14–16], as compared to more global measures of arterial stiffness via carotid-femoral PWV or other non-local techniques [14, 17]. Since subclinical arterial stiffness underlies most cardiovascular events, earlier local detection of pathological changes in arterial stiffness can improve risk stratification among those in high-risk groups, such as T1D, with and without CVD risk factors. The employment of high frequency ultrasound and ultrafast ultrasound to assess the local structure and local stiffness of the vasculature at the same location (i.e., the carotid artery) can further allow us to understand the relationship between these two measures of vascular function, and how they may be impacted by clinical variables.

In this pilot study, we evaluated hfCIMT and ultrafast ultrasound PWV (ufPWV) of the carotid artery in young adults with T1D. We hypothesized that a larger hfCIMT and higher local carotid ufPWV would be observed in T1D in relation to age and sex-matched non-diabetes controls that would be impacted by glycemia and CVD risk factors. Furthermore, we aimed to assess whether hfCIMT and ufPWV, as measures of vascular structure and stiffness, respectively, are independently associated with a T1D state, given the known early pathogenesis of CVD in T1D. This is relevant as functional changes in vascular stiffness may evolve separately from structural changes in vascular thickness, especially in younger patients who do not have a significant atherosclerotic burden.

## Methods

### Study population

This cross-sectional study included 39 participants with T1D and 25 healthy controls. It was completed at the Hospital for Sick Children (SickKids, Toronto, Canada) in 2022. All participants with T1D had participated in the Adolescent Type 1 Diabetes Cardio-Renal Intervention Trial (AdDIT, EudraCT Number: 2007-001039-72, Trial Registration Number: ISRCTN91419926) in Canada from 2009–2015, and met associated inclusion and exclusion criteria [18]. Participants were then followed as part of a longitudinal study evaluating cardio-renal-bone health from 2020–2022 (Can-SOLVE CKD). Participant recruitment for this study assessing cardiovascular structure and stiffness was conducted from the Can-SOLVE CKD cohort through convenience sampling over a 6-month period, with a 76% response rate and 39 participants recruited. No participant had declined to participate in the study, but scheduling constraints limited the response rate. Inclusion criteria were a confirmed diagnosis of type 1 diabetes according to the Diabetes Canada Clinical Practice Guidelines [19] and consent for cardiovascular assessment. Participants were excluded for a known history of cardiovascular or renal disease. Twenty-five healthy controls were enrolled based on age and sex matching. Controls derived from a prior sample of healthy controls previously used in the AdDIT/Can-SOLVE study and from advertisements, with an 80% response rate. This study conformed to the provisions of the Declaration of Helsinki and was approved by the SickKids Research Ethics Board (REB#: 1000055749; Trial Registration Number: ISRCTN91419926) and informed written consent was obtained from all participants.

### Demographics and biochemical measures

Demographic data including age, sex, and ethnicity was collected, as well as past medical history including use of concomitant medications. Anthropometrics including height, weight, body mass index (BMI), and waist circumference were collected as well [20, 21]. In addition, for T1D, details including age at diabetes onset, diabetes duration, daily total insulin dose (units/kg/day), and route of administration were collected. Information on alcohol consumption and smoking status were collected using the validated TAPS-1 Tool (Tobacco, Alcohol, Prescription medications, and other Substance), where participants chose between response options of: never, less than monthly, monthly, weekly, and daily or almost daily [22].

At the time of the study, 4-h fasting blood samples were drawn for hemoglobin A1c (A1c), glucose, creatinine, and lipid profile including low-density lipoprotein (LDL), high-density lipoprotein (HDL), total triglycerides, and cholesterol. An average A1c assessed over an extended 2-year period encompassing T1D participant involvement in the research study was also evaluated.

Blood pressure (systolic, SBP; diastolic, DBP) was measured in triplicate using an oscillometric device on the right arm (GE Healthcare, Tampa, Florida, USA) with an appropriately sized cuff. Measurements were performed with the arm at heart level after 5 min of rest with the participant seated, with the last two measures averaged to calculate blood pressure [23]. The eGFR creatinine-based equation from the Chronic Kidney Disease Epidemiology Collaboration (CKD-EPI equation) was utilized to calculated eGFR for participants [24].

### Vascular assessments

**Fig. 1:**
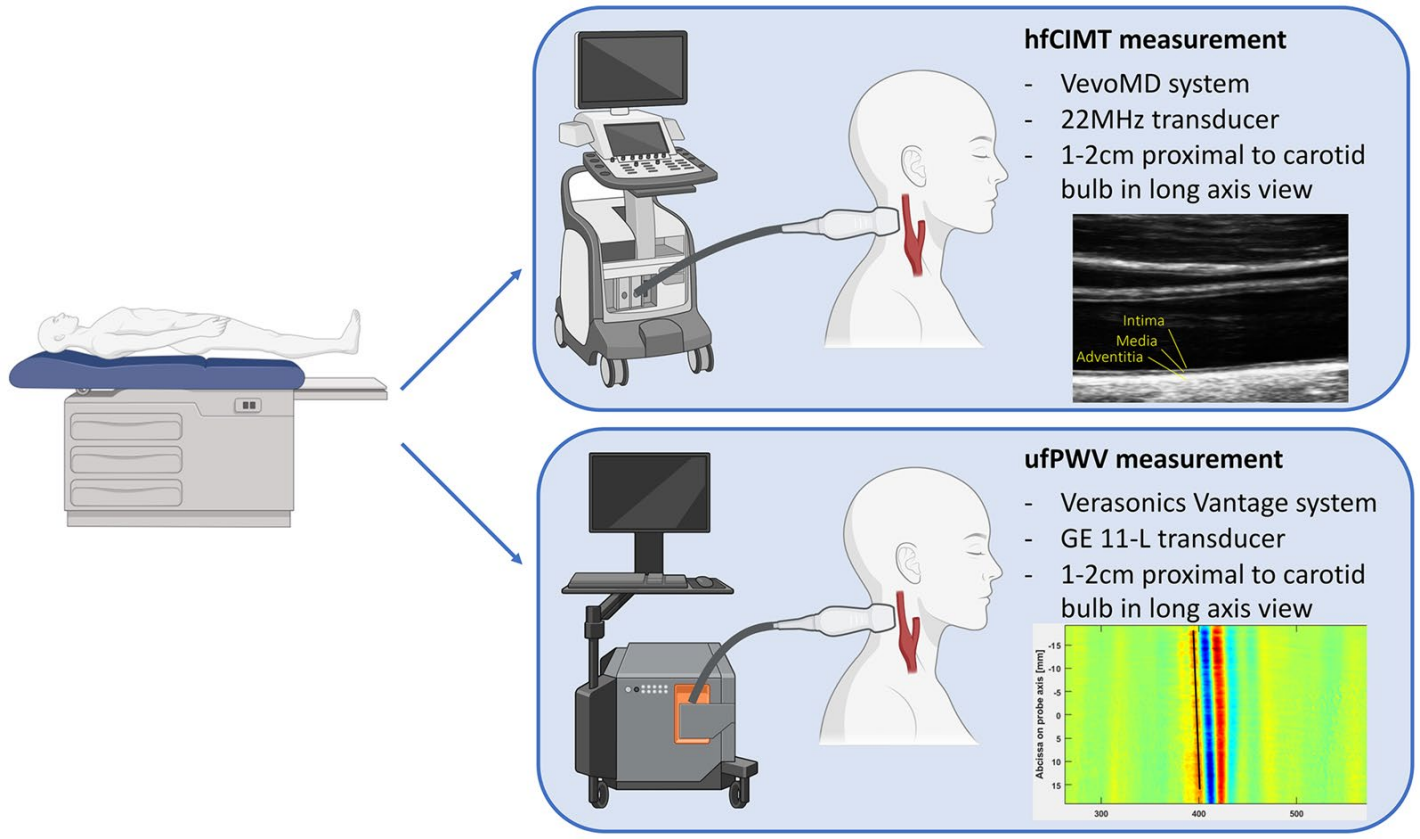
Pictorial representation of study protocol. Participants underwent recording of hfCIMT and ufPWV at the same location on the carotid artery in a supine, resting position. Inserts below in-figure text show sample raw data of hfCIMT (top) and PWN-DN (bottom). PWV is calculated from the slope of the position-time graph, as determined by radon transform, depicted by the black line at the 400s mark

Vascular structure and function were evaluated through measurement of Carotid Intima Media Thickness by High Frequency Ultrasound (hfCIMT) and Pulse Wave Velocity by ultrafast ultrasound imaging (ufPWV), as summarized in Fig. 1.

#### Carotid intima media thickness via high frequency ultrasound (hfCIMT)

hfCIMT was measured using the VevoMD ultrasound system (Fujifilm, Toronto, Canada) with a 22 MHz transducer for bilateral imaging of the common carotid artery. The common carotid arteries were imaged 1 cm proximal to the carotid bulb in a long axis view illustrating the largest lumen dimension with good distinction between structural interfaces and no compression of the blood vessel during acquisition. All image acquisitions were measured by the same operator (W.H.) [25]. The mean of three measurements were used for analysis.

#### Pulse wave velocity via ultrafast ultrasound imaging (ufPWV)

The left common carotid artery was assessed by ultrafast ultrasound imaging (UUI) using a programmable ultrafast ultrasound system (Vantage 256, Verasonics, Kirkland, WA, USA) equipped with a GE 11-L linear transducer (GE Vingmed, Horten, Norway) as previously described [26, 27]. Briefly, the probe was placed in longitudinal orientation ~ 1–2 cm proximal to the common carotid artery bifurcation, and UUI acquisition was automatically triggered by the peak of the R wave on ECG. Raw data was beamformed into In-phase/Quadrature datasets (IQ) and processed using MATLAB (The MathWorks Inc., Natick, MA, USA). Each acquisition lasted 709 ms and covered 1 cardiac cycle. ufPWV was determined offline on MATLAB using our previously published post-processing methods [26, 27]. Two pulse waves are found within a cardiac cycle: one created by the pressure wave of blood pumped out at the beginning of systole (systolic foot, PWV-SF), and one formed by the pressure differential from the aortic valve closing at the end of systole (dicrotic notch, PWV-DN). PWV-SF and PWV-DN were measured and recorded at least twice for each participant, with the average value used for each in analysis.

### Data analysis

All analysis was performed using R (version 4.2.1) [28]. Descriptive statistics were calculated for demographic, clinical, and vascular variables. Shapiro–Wilk and Q–Q plots were used to assess normality of variables. Mean and standard deviation (SD) were provided for continuous variables, otherwise median and interquartile range are reported. Frequency and percentage were reported for categorical variables. Paired students t-test, Wilcoxon rank-sum test, or chi-squared test were used to compare T1D and control groups, as appropriate. Linear regression and multiple regression were performed, as reported, including multiple regression models for evaluating hfCIMT and ufPWV in T1D versus controls. Multiple regression models were adjusted for age, sex, and BMI, as these are confounders reported to impact measured CIMT and PWV. Missing data was handled by removal. A p-value of < 0.05 was considered statistically significant.

## Results

### Demographics and biochemical measures

Participant characteristics, including demographic, anthropometric, and biochemical measures, are summarized in Table 1. T1D had a statistically significant elevation in weight, BMI, waist circumference, and height-to-waist ratio along with A1c and glucose when compared with controls. Lipid parameters and blood pressure were within reference ranges, though there were some statistically significant differences between T1D and controls.

**Table 1:** Participant characteristics

Measure	Controls *N* = *25*	T1D *N* = *39*	p-value
Age (years)	24.1 [1.9]	24.1 [1.8]	0.99
Sex			0.83
Male	16 (64)	26 (66.7)	
Female	9 (36)	13 (33.3)	
Height (cm)	175 [8]	175 [9]	0.69
Weight (kg)	**71.3 [13.0]**	**84.2 [17.1]**	**0.001**
BMI (kg/m^2^)	**22.4 {3.1}**	**26.8 {7.9}**	**0.0001**
Underweight (< 18.5 kg/m^2^)	1 (4)	0	
Normal (18.5–24.9 kg/m^2^)	18 (72)	15 (39)	
Overweight (25.0–29.9 kg/m^2^)	5 (20)	13 (33)	
Obese Class I (30.0–34.9 kg/m^2^)	1 (4)	7 (18)	
Obese Class II (35.0–39.9 kg/m^2^)	0	2 (5)	
Waist circumference (cm)	**76 [9]**	**88 [14]**	**0.0001**
Height to waist ratio	**2.33 [0.25]**	**2.04 [0.30]**	**9.9 × 10**^**–5**^
Ethnicity			0.85
Unknown/adopted	1 (4)	1 (3)	
White	13.5 (54)	22 (56)	
Black/Hispanic	2 (8)	4.75 (12)	
East Asian	4 (16)	3.25 (8)	
Arabic/Other Asian	4.5 (18)	7.25 (19)	
* South Asian/Southeast Asian*	*1.5 (6)*	*5.25 (14)*	
* Arab/Middle East*	*3 (12)*	*2 (5)*	
Indigenous	0	0.75 (2)	
Alcohol consumption*			0.32
Never	4 (16)	12 (36)	
Less than monthly	12 (48)	11 (33)	
Monthly	5 (20)	7 (21)	
Weekly	4 (16)	3 (9)	
Daily/almost daily	0	0	
T1D characteristics			
Diabetes duration (years)	–	15.1 {4.3}	–
Insulin regimen			
Injection only	–	17 (44)	–
Pump	–	22 (56)	–
A1c at testing (%, NR < 6%)	**4.9 [0.3]**	**7.4 [1.0]**	** < 2.2 × 10**^**–16**^
Average A1c (%, NR < 6%)	–	7.8 [0.8]	–
Glucose (mmol/L, NR 3.9–6.0)	**5.1 [0.3]**	**9.7 [4.0]**	** < 3.3 × 10**^**–8**^
Creatinine (µmol/L)	**79.7 [16.4]**	**68.0 [9.7]**	**0.003**
Cr males (µmol/L, NR 58–110)	**88.0 [13.0]**	**70.0 [10.2]**	** < 8.0 × 10**^**–5**^
Cr females (µmol/L, NR 46–92)	64.9 [10.0]	64.3 [7.8]	0.89
eGFR (mL/min/1.73m^2^, NR ≥ 90)	**108.0 [15.5]**	**122.4 [13.1]**	**0.0005**
ACR (mg/mmol, NR < 30)	–	0.65 {0.52}	–
Triglycerides (mmol/L, NR < 1.69)	0.81 {0.62}	0.80 {0.31}	0.77
Total cholesterol (mmol/L, NR < 5.18)	**3.86 [0.71]**	**4.37 [0.79]**	**0.011**
Friedewald LDL (mmol/L, NR < 2.59)	**1.98 [0.49]**	**2.49 [0.59]**	**0.0004**
HDL (mmol/L, NR ≥ 1.036)	1.43 [0.36]	1.37 [0.29]	0.47
SBP (mmHg, NR < 135 (130 in DM))	114 [11]	117 [10]	0.26
DBP (mmHg, NR < 85 (80 in DM))	**62 [6]**	**65 [7]**	**0.03**

Reported data shows values expressed as N (%), mean [SD], or median {inter-quartile range}. Statistically significant results (p < 0.05) indicated by bold font. Reference ranges provided in parentheses where appropriate. P-value calculated via 2-sided t-test, Wilcoxon rank-sum test, or chi-squared test, as appropriate

*T1D* type 1 diabetes, *A1c* hemoglobin A1c, *eGFR* estimated glomerular filtration rate using CKD-EPI equation, *SBP* systolic blood pressure, *DBP* diastolic blood pressure, *DM* diabetes mellitus, *NR* normal range

*N = 33 for T1D

### hfCIMT higher in type 1 diabetes

**Fig. 2:**
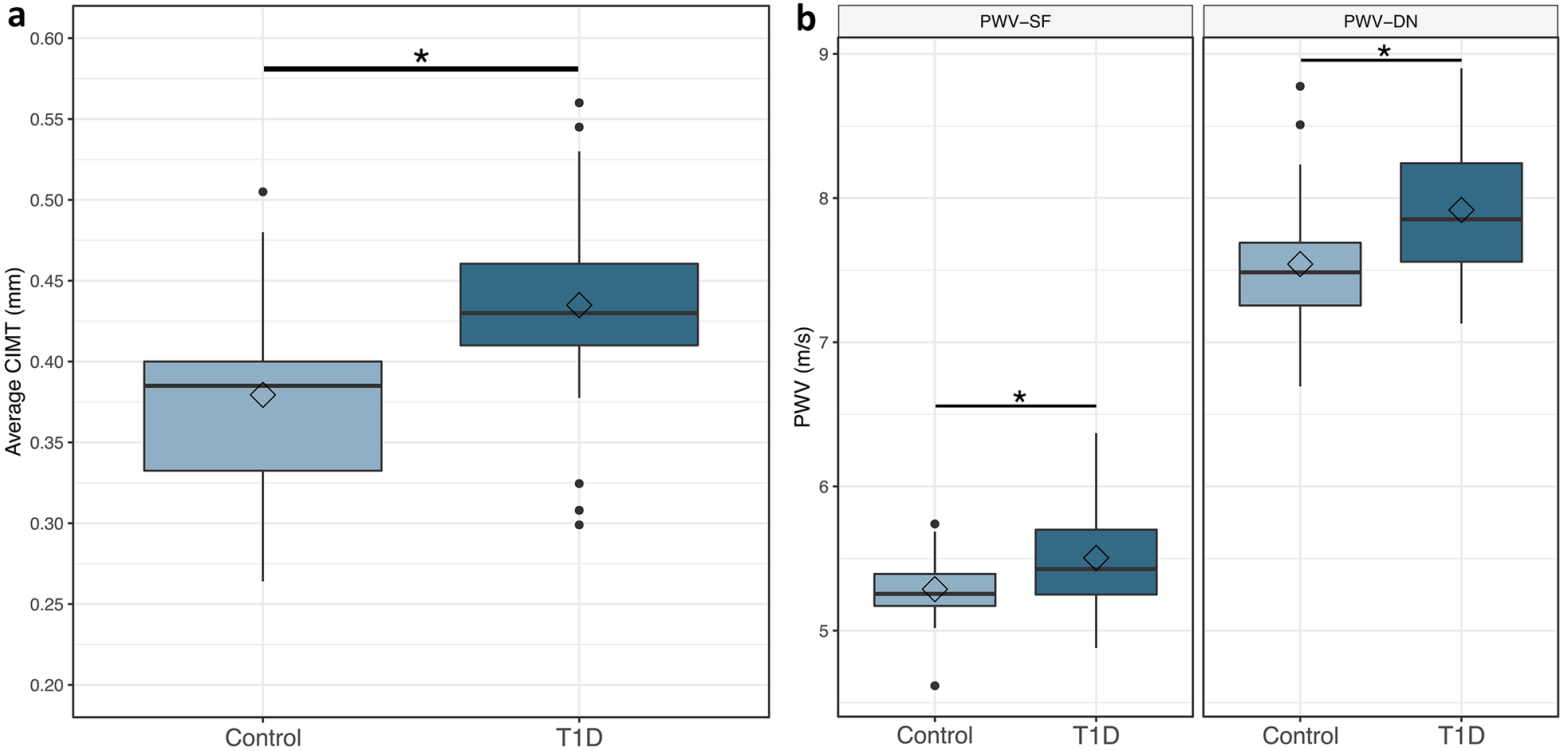
hfCIMT (**a**) and ufPWV (**b**) significantly greater in people with T1D versus healthy controls. Data represented as box-and-whisker plots. *Control* healthy controls, *T1D* people with type 1 diabetes, *CIMT* carotid intima-media thickness, *PWV* pulse wave velocity, *PWV-SF* systolic foot PWV, *PWV-DN* dicrotic notch PWV. * denotes p < 0.01

Carotid intima media thickness (hfCIMT) was significantly higher in T1D (0.379 ± 0.06 mm vs 0.435 ± 0.06 mm, p < 0.01) (Fig. 2a). After controlling for age, sex, and BMI, T1D had a 0.0465 mm increase in hfCIMT (Table 2). This corresponds to a relative percent change of 12.3% between T1D and controls. On within-group analysis of control and T1D groups, hfCIMT had no correlations to age, sex, BMI, A1c, T1D duration, total cholesterol, LDL, SBP, or DBP (data not shown).

**Table 2:** Multiple regression models for evaluating hfCIMT and ufPWV in T1D versus controls

Outcome	Exposures	Est. β-coefficient	T-statistic p-value	Adjusted R^2^	F statistic p-value
hfCIMT	Age	− 0.004	0.4	**0.16**	**0.007**
	Sex (M)	− 0.006	0.7		
	BMI	0.002	0.2		
	Class (T1D)	**0.0465**	**0.007**		
PWV-SF	Age	**0.046**	**0.04**	**0.12**	**0.02**
	Sex (M)	0.058	0.5		
	BMI	− 0.01	0.2		
	Class (T1D)	**0.267**	**0.004**		
PWV-DN	Age	0.033	0.29	**0.13**	**0.014**
	Sex (M)	− 0.028	0.8		
	BMI	− 0.01	0.3		
	Class (T1D)	**0.432**	**0.0009**		

Statistically significant results (p < 0.05) indicated by bold font

### ufPWV higher in type 1 diabetes

Pulse wave velocity at both the systolic foot (PWV-SF) and dicrotic notch (PWV-DN) was significantly increased in T1D (PWV-SF = 5.29 ± 0.23 m/s vs 5.50 ± 0.37 m/s, p < 0.01; PWV-DN = 7.54 ± 0.46 m/s vs 7.92 ± 0.41 m/s, p < 0.01) (Fig. 2b). After controlling for age, sex, and BMI, T1D had a 0.267 m/s increase in PWV-SF and 0.432 m/s increase in PWV-DN (Table 2). This corresponds to a relative percent change between T1D and controls of 5.0% and 5.7% for PWV-SF and PWV-DN, respectively. On within-group analysis of control and T1D groups, both PWV-SF and PWV-DN showed no correlation to sex, BMI, A1c, T1D duration, total cholesterol, SBP, or DBP (data not shown). PWV-SF was correlated with age (Pearson correlation coefficient r = 0.39, p = 0.01) and LDL (r = 0.38, p = 0.02) in T1D but not in controls. PWV-DN was negatively associated with LDL (r = − 0.45, p = 0.03) in controls, but had no association in T1D.

### Correlations between hfCIMT, ufPWV, and clinically-relevant variables

The correlation between hfCIMT and ufPWV was assessed using a within-group analysis of control and T1D groups. No linear correlations between hfCIMT and ufPWV parameters in both control and T1D groups were observed (Supplementary Table 1).

The impact of A1c, a measure of glycemic control, on hfCIMT and ufPWV was also assessed. A1c and class (i.e., T1D state) were highly co-linear (R^2^_adj_ = 0.69, Supplementary Fig. 1), and thus controlling for A1c alongside age, sex, and BMI demonstrated no statistical significance between hfCIMT and class (Table 3). In comparing hfCIMT models with only A1c or class that controlled for age, sex, and BMI, a marginally higher adjusted-R^2^ was observed with the A1c model compared to the class model (A1c: R^2^_adj_ = 0.18, p = 0.005, Supplementary Table 3; class: R^2^_adj_ = 0.16, p = 0.007, Table 2). For A1c, the estimated 0.0172 mm increase in hfCIMT with a 1% increase in A1c corresponds to a 0.043 mm increase in hfCIMT when accounting for the 2.5% higher A1c on average in participants with T1D (Table 1; Supplementary Table 3). This is a similar increase in hfCIMT of 0.0465 mm as seen in the class model (Table 2). This result, alongside the loss of significance when including both A1c and Class as exposures (Table 3), suggests that A1c partially mediates the relationship between class and hfCIMT, inferring that A1c can explain the higher hfCIMT observed in young adults with T1D.

**Table 3:** Multiple regression models for evaluating the impact of A1c on hfCIMT and ufPWV

Outcome	Exposures	Est. β-coefficient	T-statistic p-value	Adjusted R^2^	F statistic p-value
hfCIMT	Age	−0.004	0.40	**0.17**	**0.0095**
	Sex (M)	− 0.008	0.66		
	BMI	0.002	0.25		
	A1c	0.011	0.29		
	Class (T1D)	0.023	0.44		
PWV-SF	Age	0.043	0.061	**0.10**	**0.049**
	Sex (M)	0.04	0.69		
	BMI	− 0.01	0.097		
	A1c	0.02	0.76		
	Class (T1D)	0.23	0.14		
PWV-DN	Age	0.03	0.35	**0.11**	**0.037**
	Sex (M)	− 0.03	0.79		
	BMI	− 0.01	0.23		
	A1c	− 0.02	0.78		
	Class (T1D)	**0.479**	**0.03**		

Statistically significant results (p < 0.05) indicated by bold font

Despite the high co-linearity between A1c and class, PWV-DN remained significantly associated with the T1D state when controlling for age, sex, BMI, and A1c; specifically, participants with T1D had an increase in PWV-DN of 0.479 m/s (Table 3). Indeed, when employing a PWV-DN model with only A1c or class that controlled for age, sex, and BMI, a higher adjusted-R^2^ was observed with the class model compared to the A1c model [class: R^2^_adj_ = 0.13, p = 0.014 (Table 2) versus A1c: R^2^_adj_ = 0.05, p = 0.14 (Supplementary Table 3)]. Despite seeing an increase in PWV-DN with higher A1c, the model with A1c was not significant (p = 0.14; Supplementary Table 3), inferring that A1c does not explain the higher PWV-DN observed in young adults with T1D.

Similar models with PWV-SF had no significance; controlling for A1c alongside age, sex, and BMI demonstrated no statistical significance between PWV-SF and class (Table 3). While the PWV-SF model with class had a higher adjusted-R^2^ than the model with A1c when controlling for age, sex, and BMI, the model with A1c was non-significant (class: R^2^_adj_ = 0.12, p = 0.019, Table 2; A1c: R^2^_adj_ = 0.06, p = 0.06, Supplementary Table 3). Given this, we cannot infer how A1c impacts the relationship between PWV-SF and class in our study population.

Finally, evaluations of blood pressure and lipid parameters demonstrated they had no impact on the measured hfCIMT or ufPWV (see Supplemental Materials for more information).

## Discussion

In this study, high frequency ultrasound and ultrafast ultrasound were used to measure CIMT and PWV of the common carotid artery in T1D and healthy controls. We observed that young adults with T1D had a significantly higher carotid artery hfCIMT and ufPWV as compared to healthy controls. Furthermore, we found A1C-measured glycemia was an important factor to explain the difference in measured hfCIMT between T1D and controls, but it was not an important factor to explain the difference in measured ufPWV.

Using high resolution imaging, hfCIMT and ufPWV, we demonstrate increases in vascular structure and stiffness that have previously been shown through traditional CIMT and carotid-femoral PWV, and provide important insights into the relationship between these measures of vascular structure and function in T1D. Studies have demonstrated an increased CIMT and carotid-femoral PWV in T1D [29], and a recent meta-analysis on the topic concluded that youth with T1D have an increased CIMT and carotid-femoral PWV as compared to healthy controls [30]. The previously reported difference in CIMT of 0.03 mm from a meta-analysis of 20 studies on T1D [30] is similar to the difference seen in hfCIMT for our cohort, where we report a 0.0465 mm increase after controlling for age, sex and BMI (Table 2). The discrepancy in hfCIMT between T1D and healthy controls reported in this study compared to the prior metanalysis likely relates to clinical characteristics of the study population, being older in age (24 years vs. 15 years) [30] with longer T1D duration (16.1 years vs 6.9 years) [30], in addition to the higher resolution ultrasound images allowing for a more precise measurement of CIMT [12].

While significant differences were observed in T1D youth using both hfCIMT and ufPWV, significant correlations between these measures were not present in T1D youth or healthy controls. These are likely related to the interplay of age and developmental stage on vascular structure and function as well as CVD progression. Theoretically, PWV is proportional to arterial elasticity (Young’s modulus) and thickness and inversely proportional to arterial diameter [31]. In older adults, PWV has been observed to be positively associated with CIMT [31, 32]; for example, in a cross-sectional study of adults (mean age 56 years old), PWV was correlated to CIMT, and the two remained associated after adjusting for age and blood pressure [32]. However, studies also describe increased carotid PWV despite a normal CIMT in people with hyperlipidemia and T1D as well as in healthy individuals [33–36]. This lack of a direct link between CIMT and PWV is likely related to other factors impacting PWV, including variations in vascular elasticity, the dependence of PWV on blood pressure, and, when considering local ultrafast PWV, the differences found between measured PWV-SF (systolic foot) and PWV-DN (dicrotic notch) that gauge arterial stiffness at distinct pressure states in the cardiac cycle [34, 37, 38].

The younger age of participants in this study is also important, as the evolution of vascular thickening and stiffening with aging is influenced by atherosclerotic processes [39]. These processes would be premature in a younger adult population, where an increased CIMT is more likely linked to endothelial dysfunction and the epigenetic and environmental changes associated with diabetes [40, 41]. Furthermore, studies in diabetes have suggested that functional changes measured via ufPWV may evolve separately from structural changes measured via CIMT [34]. Altogether, these factors are important considerations when assessing the relationship between local ultrafast PWV and CIMT, and when considering that measures of functional and structural change in local vasculature should be evaluated independently.

A notable aspect of our study population was that the average HbA1c in the T1D group was at the lower end of the range that we have previously reported from our larger follow-up cohort (Mean A1C: 8.18 ± 1.03%) [42] and HbA1c levels typically found in the general populous of people with T1D. While the HbA1c levels in our cohort were 7.4% (Table 1), the majority of young adults with T1D have a HbA1c > 7.5%, with ~ 40% having a HbA1c > 9.0% [43]. The good glycemic control in our cohort is most likely attributed to selection bias through recruitment in our study, as well as recent clinical trends towards lower HbA1c levels with increased use of technology [44]. Despite this, we still saw a statistically significant difference in hfCIMT and ufPWV in people with T1D with better glycemic control.

While there was no CVD in this young cohort, there were clinical features of greater cardiovascular risk, including higher BMI and abnormal lipids in the T1D group (Table 1). Despite evaluating these traditional confounders in our analysis, we observed that A1c and T1D status primarily resulted in the differences in hfCIMT and ufPWV seen in T1D youth. While we cannot rule out the effects of lipids or blood pressure, especially later in life and in the pathogenesis of T1D, we did not observe a significant impact of these parameters at this stage on these measures.

Interestingly, A1c, as a measure of glycemic control, had differential impacts on the relationship between hfCIMT or ufPWV and T1D status. These findings raise the possibility that glycemia may impact early vascular structure and function differently and highlight hfCIMT and ufPWV as distinctive CVD measures. While A1c has been correlated to both CIMT and PWV in people with longstanding diabetes, this may not hold true in the general adult population, where A1c has been shown to be associated with PWV but not CIMT [45]. In our cohort, we found that A1c was a partial mediator of the relationship between hfCIMT and T1D status (Table 2, 3; Supplementary Table 3), implying that higher A1c contributes to the increased hfCIMT in people with T1D. In contrast, A1c was found to be a confounder of the relationship between PWV-DN and T1D status (Table 2, 3; Supplementary Table 3), indicating that there are additional factors beyond A1c associated with the higher ufPWV in our T1D cohort. It is likely that other aspects of the diabetic state are driving the functional vascular changes in T1D. These include dynamic changes in local vasoactive mediators (i.e., growth factors, nitric oxide synthase), inflammation due to hyperglycemia, effects of hyperglycemia on advanced glycation end products (AGE) and collagen glycation, epigenetics, and oxidative damage of endothelial cells, which have been cited to play a role in the pathogenesis of CVD in T1D [40, 41, 46–50]. Therefore, the diabetic state alters vascular structure and functional stiffness to increase cardiovascular risk in T1D, changes that progress at different rates and are impacted by different aspects of the diabetic state. Measurement of CIMT and PWV together allows for an assessment of changes in both the structure and functional stiffness of the vasculature in people with T1D, which may enhance assessment of vascular risk and guide intervention to reduce the burden of cardiovascular disease in this population.

### Limitations

This study had limitations. T1D participants had better control and management of their glycemic levels than T1D of larger registry-based cohorts [43]; despite this, we still found significant changes in hfCIMT and ufPWV. Secondly, we did not measure the body composition or lean body mass of participants. Higher lean body mass has been associated with higher CIMT in healthy individuals [51] and may be a cause for variation in cardiovascular parameters not addressed in this analysis. Further, this evaluation includes a small sample of T1D and healthy controls. This study was a pilot study with new and analysis-intensive methods that are not yet widely clinically available. Though the small sample size limited statistical power in analyses, differences were observed with consistent directionality of the data.

## Conclusions

We report that young adults with type 1 diabetes had a significantly higher carotid artery hfCIMT and ufPWV as compared to healthy controls. Clinical manifestations of CVD develop insidiously and appear late in CVD pathogenesis, and opportunities for prevention strategies need to be implemented at an earlier, pre-clinical phase. The evaluation of advanced and novel ultrasound techniques using both high frequency ultrasound and ultrafast ultrasound imaging is important for measuring subclinical changes in vascular structure and function in youth with T1D who are at high risk of CVD. Future work using these high resolution, ultrasensitive measures should be investigated in larger cohorts to further evaluate the interplay of vascular structure and function in the progression of CVD in T1D.

### Supplementary Information

Supplementry file1 (PDF 479 kb)

## Data Availability

The datasets generated and/or analysed during the current study are not publicly available but reasonable requests may be forwarded to the Senior author for review (F.H.M).

## Abbreviations

A1c: Hemoglobin A1c
CIMT: Carotid intima media thickness
cfPWV: Carotid-femoral pulse wave velocity
CVD: Cardiovascular disease
HFS: High frequency ultrasound
hfCIMT: Carotid intima media thickness measured by high frequency ultrasound
PWV: Pulse wave velocity
PWV-DN: Ultrafast pulse wave velocity at the dicrotic notch
PWV-SF: Ultrafast pulse wave velocity at the systolic foot
T1D: Type 1 diabetes
UUI: Ultrafast ultrasound imaging
ufPWV: Ultrafast pulse wave velocity (measured by ultrafast ultrasound imaging)
