# Supplementary material for: Abnormal vascular thickness and stiffness in young adults with type 1 diabetes: new insights from cutting-edge ultrasound modalities
Source: Cardiovasc Diabetol. 2024 May 24;23:178. doi: 10.1186/s12933-024-02280-5 (PMC11127355; doi:10.1186/s12933-024-02280-5)
Supplement: Supplementary file 1 — Supplementry file1 (PDF 479 kb) [file 12933_2024_2280_MOESM1_ESM.pdf]

SUPPLEMENTAL MATERIALS

Supplemental Results

Direct linear models to assess multi-collinearity

Direct linear models were employed to assess collinearity (**Suppl. Figure 1**). Strong, significant associations were found between class (i.e., T1D or controls) and A1c (R^2_{adj} = 0.69). Additional significant associations were also found between PWV-SF and PWV-DN (R^2_{adj} = 0.35) and LDL and cholesterol (R^2_{adj} = 0.78). There were also weaker but significant correlations between other lipid parameters, such as between triglycerides and cholesterol (R^2_{adj} = 0.16) and HDL and cholesterol (R^2_{adj} = 0.14). Notably, there was no significant association between ufPWV and either SBP or DBP, indicating the measured ufPWV was not influenced by blood pressure in our cohort of young adults.

Evaluating Correlation between hfCIMT and ufPWV

To further evaluate the relationship between hfCIMT and ufPWV, the impact of hfCIMT on the relationship between ufPWV (PWV-SF/PWV-DN) and class (i.e., T1D or controls) was determined, and we similarly evaluated the impact of ufPWV on the relationship between hfCIMT and class. hfCIMT or ufPWV were included as an additional exposure variable in multiple regression models with an outcome variable of ufPWV or hfCIMT, respectively, while controlling for age, sex, and BMI. When either hfCIMT or ufPWV were included in multiple regression models, they did not have significant associations and did not impact the relationship between class and ufPWV or hfCIMT, respectively (**Suppl. Table 2**).

Correlations of blood pressure and lipid parameters with hfCIMT and ufPWV

In simple linear models that controlled for age, sex, and BMI, there was no association found between hfCIMT or ufPWV and blood pressure (SBP or DBP) (data not shown). Further, when treated as an exposure alongside class, SBP and DBP had no significant predictive value in almost all instances, instead serving as a statistical confounder to minimize the strength of the model (data not shown). Like blood pressure, simple linear models controlling for age, sex, and BMI found no association between hfCIMT or ufPWV and lipid parameters (LDL, HDL, TG, total cholesterol) and had poor predictive value (**Suppl. Table 4**). Moreover, when LDL was included in models of association between class and hfCIMT or ufPWV, it served as a statistical confounder with no significant predictive value (**Suppl. Table 5**). Since both the lipid parameters and blood pressure did not have significant associations and did not improve the power of the regression models, they were not included in other analyses.

Supplemental Figures and Tables

R^2	Class	HbA1c	CIMTAVE	PWV-SF	PWV-DN	Age	Sex	BMI	LDL	Chol	HDL	TG	SBP	DBP
ClassT1D		0.69	0.17	0.09	0.14	-0.02	NA	0.17	0.16	0.08	-0.01	-0.01	0.04	0.07
HbA1c	0.69		0.18	0.05	0.07	-0.02	0.00	0.16	0.08	0.03	0.00	-0.01	0.00	0.03
CIMTAVE	0.17	0.18		-0.02	-0.01	-0.01	-0.02	0.08	0.04	0.01	-0.01	-0.02	0.00	-0.01
PWV-SF	0.09	0.05	-0.02		0.35	0.03	-0.01	-0.02	0.09	0.04	0.00	-0.01	0.00	-0.01
PWV-DN	0.14	0.07	-0.01	0.35		0.00	-0.01	-0.01	0.02	0.00	0.00	0.00	-0.01	-0.01
Age	-0.02	-0.02	-0.01	0.03	0.00		0.01	0.00	0.09	0.11	0.00	0.00	-0.02	-0.02
SexM	NA	0.00	-0.02	-0.01	-0.01	0.01		-0.01	-0.01	-0.02	0.10	0.01	0.17	0.00
BMI	0.17	0.16	0.08	-0.02	-0.01	0.00	-0.01		0.09	0.05	0.05	0.04	0.07	-0.01
LDL	0.16	0.08	0.04	0.09	0.02	0.09	-0.01	0.09		0.78	0.03	-0.01	0.02	0.04
Chol	0.08	0.03	0.01	0.04	0.00	0.11	-0.02	0.05	0.78		0.14	0.16	0.00	0.02
HDL	-0.01	0.00	-0.01	0.00	0.00	0.00	0.10	0.05	0.03	0.14		0.09	0.07	-0.02
TG	-0.01	-0.01	-0.02	-0.01	0.00	0.00	0.01	0.04	-0.01	0.16	0.09		0.02	-0.01
SBP	0.04	0.00	0.00	0.00	-0.01	-0.02	0.17	0.07	0.02	0.00	0.07	0.02		0.22
DBP	0.07	0.03	-0.01	-0.01	-0.01	-0.02	0.00	-0.01	0.04	0.02	-0.02	-0.01	0.22	

Supplemental Figure 1: Direct Linear Models to assess for multi-co-linearity. Highlighted R^2_{adj} values have a statistically significant p-value <0.05.

Supplemental Table 1: **Correlation between hfCIMT and ufPWV for within-group comparisons.** Linear models evaluated as shown for controls (N=25) or T1D (N=39) groups. R^2 indicates the strength of the correlation; p-value indicates the statistical significance of the correlation.

Class	Model	R^2_{adj}	p-value
Controls	hfCIMT ~ PWV-SF	0.060	0.13
	hfCIMT ~ PWV-DN	-0.043	0.92
T1D	hfCIMT ~ PWV-SF	0.014	0.49
	hfCIMT ~ PWV-DN	0.012	0.24

Supplemental Table 2: **Multiple regression models for assessing the relationship between hfCIMT and ufPWV.** Statistically significant results ($p<0.05$) are in bold.

**Outcome**	**Exposures**	Est. β-coefficient	T-statistic p-value	**Adjusted R^2**	**F statistic p-value**
hfCIMT	Age	-0.002	0.58	**0.16**	**0.011**
	Sex (M)	-0.004	0.80		
	BMI	0.002	0.26		
	PWV-SF	-0.02	0.33		
	Class (T1D)	**0.0527**	**0.0047**		
hfCIMT	Age	-0.003	0.46	**0.15**	**0.013**
	Sex (M)	-0.006	0.72		
	BMI	0.002	0.24		
	PWV-DN	-0.01	0.50		
	Class (T1D)	**0.0517**	**0.0070**		
PWV-SF	Age	0.05	0.051	**0.12**	**0.031**
	Sex (M)	0.06	0.53		
	BMI	-0.008	0.37		
	hfCIMT	-0.71	0.33		
	Class (T1D)	**0.292**	**0.0039**		
PWV-DN	Age	0.03	0.35	**0.12**	**0.032**
	Sex (M)	-0.02	0.89		
	BMI	-0.01	0.31		
	hfCIMT	-0.69	0.50		
	Class (T1D)	**0.467**	**0.0010**		

Supplemental Table 3: **Nested multiple regression models for evaluating the impact of A1c on hfCIMT and ufPWV.** Statistically significant results ($p<0.05$) are in bold.

**Outcome**	**Exposures**	Est. β-coefficient	T-statistic p-value	**Adjusted R^2**	**F statistic p-value**
hfCIMT	Age	-0.004	0.38	**0.18**	**0.0053**
	Sex (M)	-0.010	0.56		
	BMI	0.002	0.20		
	A1c	**0.017**	**0.0050**		
PWV-SF	Age	0.04	0.073	0.08	0.061
	Sex (M)	0.009	0.91		
	BMI	-0.01	0.15		
	A1c	**0.0795**	**0.015**		
PWV-DN	Age	0.03	0.42	0.05	0.14
	Sex (M)	-0.09	0.48		
	BMI	-0.01	0.40		
	A1c	**0.11**	**0.015**		

Supplemental Table 4: **Multiple regression models for evaluating the impact of lipid parameters on hfCIMT and ufPWV, irrespective of class.** Statistically significant results ($p<0.05$) are in bold.

**Outcome**	**Exposures**	Est. β-coefficient	T-statistic p-value	**Adjusted R^2**	**F statistic p-value**
hfCIMT	Age	-0.007	0.16	0.07	0.087
	Sex (M)	-0.008	0.64		
	BMI	0.003	0.074		
	LDL	0.02	0.14		
PWV-SF	Age	0.03	0.28	0.08	0.062
	Sex (M)	0.05	0.54		
	BMI	-0.004	0.64		
	LDL	**0.17**	**0.025**		
PWV-DN	Age	0.01	0.71	-0.03	0.65
	Sex (M)	-0.04	0.76		
	BMI	0.001	0.90		
	LDL	0.13	0.24		
hfCIMT	Age	-0.006	0.24	0.06	0.11
	Sex (M)	-0.005	0.76		
	BMI	**0.0036**	**0.030**		
	Cholesterol	0.01	0.37		
PWV-SF	Age	0.03	0.27	0.03	0.20
	Sex (M)	0.06	0.54		
	BMI	-0.004	0.66		
	Cholesterol	0.09	0.10		
PWV-DN	Age	0.02	0.64	-0.04	0.80
	Sex (M)	-0.03	0.80		
	BMI	0.003	0.79		
	Cholesterol	0.06	0.45		
hfCIMT	Age	-0.004	0.38	0.05	0.15
	Sex (M)	-0.007	0.73		
	BMI	**0.004**	**0.016**		
	HDL	-0.003	0.91		
PWV-SF	Age	0.04	0.11	0.007	0.36
	Sex (M)	0.09	0.37		
	BMI	0.002	0.85		
	HDL	0.15	0.30		
PWV-DN	Age	0.02	0.51	-0.03	0.72
	Sex (M)	0.009	0.95		
	BMI	0.008	0.50		
	HDL	0.21	0.32		
hfCIMT	Age	-0.004	0.40	0.05	0.15
	Sex (M)	-0.005	0.79		
	BMI	**0.004**	**0.013**		
	TG	-0.004	0.70		
PWV-SF	Age	0.045	0.063	0.002	0.40

	Sex (M)	0.07	0.46		
	BMI	0.001	0.91		
	TG	-0.05	0.37		
PWV-DN	Age	0.03	0.35	-0.03	0.66
	Sex (M)	-0.008	0.95		
	BMI	0.008	0.49		
	TG	-0.096	0.26		

Supplemental Table 5: **Multiple regression models for evaluating the impact of LDL on the relationship of hfCIMT and ufPWV with class**. Statistically significant results ($p<0.05$) are in bold.

**Outcome**	**Exposures**	Est. β-coefficient	T-statistic p-value	**Adjusted R^2**	**F statistic p-value**
hfCIMT	Age	-0.005	0.31	**0.14**	**0.021**
	Sex (M)	-0.007	0.67		
	BMI	0.002	0.29		
	LDL	0.008	0.58		
	Class (T1D)	**0.0424**	**0.02**		
PWV-SF	Age	0.04	0.12	**0.15**	**0.014**
	Sex (M)	0.06	0.46		
	BMI	-0.01	0.22		
	LDL	0.10	0.21		
	Class (T1D)	**0.223**	**0.023**		
PWV-DN	Age	0.03	0.31	**0.12**	**0.032**
	Sex (M)	-0.02	0.84		
	BMI	-0.01	0.34		
	LDL	-0.01	0.92		
	Class (T1D)	**0.438**	**0.0021**		
